# In Streptococcus thermophilus, Ammonia from Urea Hydrolysis Paradoxically Boosts Acidification and Reveals a New Regulatory Mechanism of Glycolysis

**DOI:** 10.1128/spectrum.02760-21

**Published:** 2022-04-25

**Authors:** Stefania Arioli, Giulia Della Scala, Anđela Martinović, Leonardo Scaglioni, Stefania Mazzini, Federica Volonté, Martin Bastian Pedersen, Diego Mora

**Affiliations:** a Department of Food, Environmental, and Nutritional Sciences (DeFENS), University of Milan, Milan, Italy; b Sacco System, Cadorago, Italy; Ohio State University

**Keywords:** *Streptococcus thermophilus*, glycolysis, homolactic fermentation, ammonia, urease, phosphofructokinase

## Abstract

Streptococcus thermophilus is widely used in the dairy industry for the manufacturing of fermented milk and cheeses and probiotic formulations. S. thermophilus evolved from closely phylogenetically related pathogenic streptococci through loss-of-function events counterbalanced by the acquisition of relevant traits, such as lactose and urea utilization for the adaptation to the milk environment. In the context of regressive evolution, the urease gene cluster accounts for 0.9% of the total coding sequence belonging to known functional categories. The fate of ammonia and carbon dioxide derived by urea hydrolysis in several biosynthetic pathways have been depicted, and the positive effect of urease activity on S. thermophilus growth fitness and lactic acid fermentation in milk has been already addressed by several authors. However, the mechanistic effect of urea hydrolysis on the energetic metabolisms of S. thermophilus is still unclear. This study aimed to assess the effect of urease activity on the growth and energy metabolism of Streptococcus thermophilus in milk. In milk, ^13^C-urea was completely hydrolyzed in the first 150 min of S. thermophilus growth, and urea hydrolysis was accompanied by an increase in cell density and a reduction in the generation time. By using energetically discharged cells with gene transcription and translation blocked, we showed that in the presence of fermentable carbon sources, urease activity, specifically the production of ammonia, could dramatically boost glycolysis and, in cascade, homolactic fermentation. Furthermore, we showed that ammonia, specifically ammonium ions, were potent effectors of phosphofructokinase, a key glycolytic enzyme.

**IMPORTANCE** Finding that ammonia-generating enzymes, such as urease, and exogenous ammonia act on phosphofructokinase activity shed new light on the regulatory mechanisms that govern glycolysis. Phosphofructokinase is the key enzyme known to exert a regulatory role on glycolytic flux and, therefore, ammonia as an effector of phosphofructokinase acts, in cascade, modulating the glycolytic pathway. Apart from S. thermophilus, due to the high conservation of glycolytic enzymes in all branches of the tree of life and being aware of the role of ammonia as an effector of phosphofructokinase, we propose to reevaluate the physiological role of the ammonia production pathways in all organisms whose energy metabolism is supported by glycolysis.

## INTRODUCTION

Streptococcus thermophilus is a lactic acid bacterium that shows some peculiar characteristics that makes it unique among the other lactic acid bacterial species. Due to its safe use in food production over the years, S. thermophilus was granted “generally recognized as safe” (GRAS) status in the USA and “qualified presumption of safety” (QPS) status in the European Union (1). Genome analysis and comparison confirmed the safety status and provided insights into adaptive evolutionary mechanisms that lead to speciation from human commensal and human pathogens. In this context, the acquisition of new traits, such as the utilization of lactose, intriguingly correlated with the appearance of mammals on earth in the late Paleocene between 65 and 23 mya, helping S. thermophilus adapt to the dairy environment. Therefore, as can be deduced from Bolotin et al. (2), the emergence of milk represents the breaking point at which the evolutionary path of S. thermophilus diverged from that of human commensal streptococci. The energy metabolism of S. thermophilus is based on the homolactic fermentation of the milk sugar lactose, which enters the cell thanks to an extremely efficient lactose transport, the permease LacS. LacS catalyzes the heterologous exchange of lactose for intracellularly formed galactose as well as proton motive force (Δp)-driven lactose uptake (3). Galactose is generated by the activity of a β-galactosidase inside the cell. Therefore, galactose is excreted in equimolar amounts with lactose transported. The glucose moiety of lactose is catabolized by glycolysis and homolactic fermentation. l-lactate is the major fermentation end product from sugar metabolism (>95%) with the production of small amounts of alternative fermentation end products, such as acetoin, acetaldehyde, and formate (4). Nevertheless, except for the reduction of pyruvate to l-lactic acid by lactate dehydrogenase, no other alternative for NAD^+^ regeneration is possible (4, 5). This relatively simple metabolism of S. thermophilus has been studied by several to develop or select mutants able to also use the galactose moiety of lactose and to biochemically characterize its unique lactose transport. However, the regulatory mechanisms that govern the energy metabolism of S. thermophilus have been poorly investigated (6–9).

Among lactic acid bacteria of dairy interest, S. thermophilus is the only species harboring an active urease. The role of urease activity in S. thermophilus in modulating environmental pH and its involvement in the biosynthesis of aspartate, glutamine, and arginine have already been investigated (10–14). Moreover, it was also reported that hydrolysis of urea increases the catabolic yield of S. thermophilus by modulating the intracellular pH and increasing the activity of β-galactosidase, glycolytic enzymes, and lactate dehydrogenase (7). More recently, it was reported that urease deficiency severely decreased acidification rates and bacterial counts in milk (14).

According to Yamauki et al. (14), urease is important for the growth of S. thermophilus mainly through NH_3_ supply. Nevertheless, it is still controversial whether the positive effect on S. thermophilus metabolism of ammonia released by urease is due to ammonia as a nitrogen source, ammonia as an alkalizing agent, or both.

To better understand the role of urease and ammonia on bacterial growth and homolactic fermentation of S. thermophilus, we decided to monitor the first 3 h of growth in milk. Moreover, we used energetically discharged cells blocked in the transcription and translation machinery by rifamycin and chloramphenicol supplementation to demonstrate that urease activity, specifically urea-derived NH_3_, boosts homolactic fermentation through a transcription- and translation-independent mechanism.

## RESULTS

### Urea hydrolysis boosted the growth rate and homolactic fermentation of S. thermophilus in milk.

**FIG 1:**
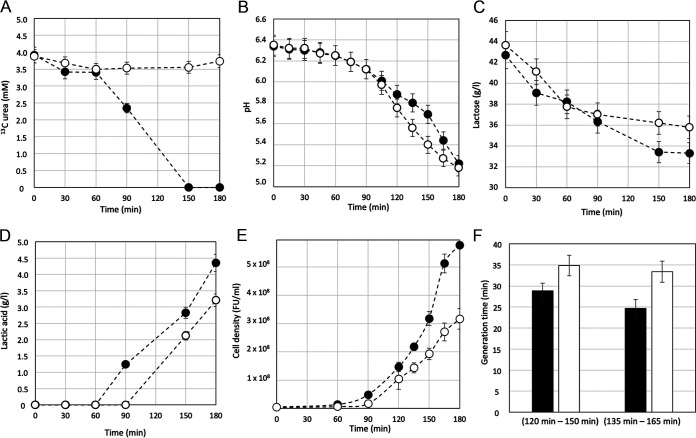
S. thermophilus urea hydrolysis, growth rate, and homolactic fermentation in milk. Monitoring ^13^C urea consumption (A), acidification (B), lactose consumption (C), lactic acid production (D), cell density (E), and generation time (F) during the growth of S. thermophilus MIM13 in skimmed milk with (open circle) or without (black circle) supplementation of flurofamide as a urease inhibitor. The generation time (F) was calculated between 120 min and 150 min and between 135 min and 165 min. Error bars represent the standard deviation calculated on three experimental replicates.

Milk contains different amounts of urea from one batch to another (ranging from 3 to 6 mM). Therefore, we supplement skimmed milk, with 4 mM ^13^C urea (15). Monitoring of ^13^C urea consumption (Fig. 1A) revealed that S. thermophilus MIM13 started to hydrolyze urea between 60 and 90 min from the inoculum when the milk pH was between 6.25 (± 0.1) and 6.12 (± 0.1) (Fig. 1B). At 150 min from the inoculum, ^13^C urea was not detectable. It was, therefore, considered completely hydrolyzed (Fig. 1A). The start of urea hydrolysis was accompanied by an expected buffering effect of urea-generated ammonia toward the lactic acid produced by homolactic fermentation. Alkalization due to ammonia release, which reduced the milk acidification rate, was detected starting 90 to 120 min from the inoculum time (Fig. 1B), and it was accompanied by an increase in lactose consumption and lactic acid production compared with the culture condition where milk was supplemented with the urease inhibitor flurofamide (Fig. 1C and D). It is worth mentioning that flurofamide at the concentration used did not affect the growth of S. thermophilus in a medium devoid of urea (16). The overall data collected in the first 180 min of growth in milk highlighted that (i) urea hydrolysis started when the pH of the milk could not be considered critical for the growth of S. thermophilus (Fig. 1B), (ii) inhibition of urea hydrolysis by flurofamide resulted in decreased lactose consumption (Fig. 1C) and homolactic fermentation (Fig. 1D), and (iii) inhibition of urea hydrolysis by flurofamide resulted in a decrease in S. thermophilus growth fitness in terms of cell density (Fig. 1E) and generation time (Fig. 1F).

### Ammonia boosted acidification in nongrowing S. thermophilus cells.

**FIG 2:**
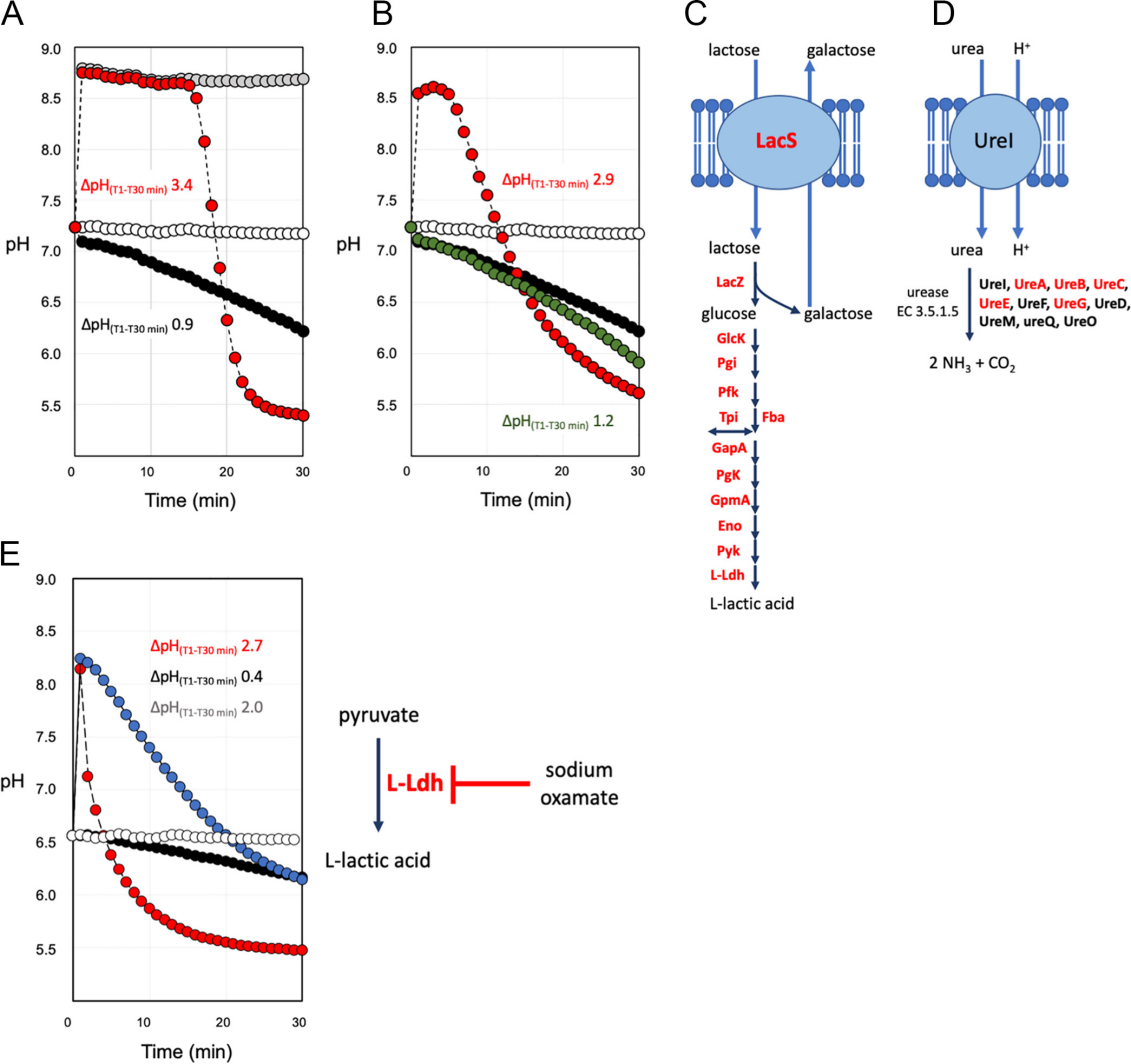
Activation of homolactic fermentation in EdCs. EdCs fed l4 mM lactose and NH_3_ (A) or urea (B) (red circles). EdCs treated with 2 mM NH_3_ (gray circles). EdCs, (white circles). EdCs treated with lactose (black circles). EdCs activated with lactose, urea, and flurofamide (green circles). EdCs treated with lactose, NH_3_, and sodium oxamate (blue circle). For (A, B, and E), ΔpH_(T1-T30 min)_ values are reported. (C) Transport proteins and enzymes involved in lactose metabolism are reported. Transport proteins and enzymes detected in the proteome of EdCs are written in red. LacS, lactose permease. LacZ, β-galactosidase. GlcK, glucose kinase. Pgi, glucose-6-phosphate isomerase. Pfk, 6-phosphofructokinase. Tpi, triosephosphate isomerase. Fba, fructose-bisphosphate aldolase. GapA, glyceraldehyde-3-phosphate dehydrogenase. Pgk, phospho-glycerate kinase. GpmA, phosphoglycerate mutase. Eno, enolase. Pyk, pyruvate kinase. l-ldh, l-lactate dehydrogenase. (D) Schematic representation of urease activity. Proteins involved in urease biosynthesis are reported. Proteins detected in the proteome of EdCs are written in red. Data are reported as the average of three replicates. The standard deviation was always below 2% for the measured pH.

The role of urea and/or ammonia as enhancers of S. thermophilus acidification was further investigated in nongrowing S. thermophilus cells. These biomasses were prepared from exponential growing cells, collected, and immediately suspended in a saline solution containing chloramphenicol and rifamycin, maintained in that solution, and exposed to urea as described by Arioli et al. (7). This cell preparation has been called energetically discharged (EdCs) because during urea hydrolysis the intracellular ATP concentration showed a peak in the first 8 min of treatment and then a rapid decrease (7). S. thermophilus EdCs suspension contains rifamycin and chloramphenicol, which act on transcription and translation, to exclude the effect of any regulatory mechanisms on measured metabolic parameters. Moreover, the use of nongrowing cells should also exclude the effect of ammonia as a nitrogen source in anabolic pathways. Due to the simplicity of the energy metabolism of S. thermophilus, homolactic fermentation can be followed by monitoring the medium acidification when feeding cells lactose. The results obtained showed that in the presence of lactose, acidification was strongly increased when ammonia or urea was supplemented (Fig. 2A and B). The proteomic data, carried out on the EdC, confirmed the presence of urease subunits and accessory proteins, and all enzymes of lactose catabolism (Fig. 2C and D). When urea was supplemented, but urease activity was inhibited by flurofamide, acidification was significantly reduced (Fig. 2B). When EdCs were treated with sodium oxamate, a pyruvate analog acting as a moderate inhibitor of lactate dehydrogenase, the acidification stimulated by lactose and ammonia was severely delayed (Fig. 2E) (17). Ammonium chloride was much less effective in promoting acidification (Fig. S1A). This could be explained by the fact that ammonia was a weak base (pK_b_ 4.75), giving alkaline pH in an aqueous solution. At physiological pH values of 7 to 7.5, ammonia (NH_3_) and ammonium (NH_4_^+^) rapidly reached equilibrium, with NH_4_^+^ being the dominant form. Ammonia as a small nonprotonated and lipophilic molecule can rapidly diffuse across the cell membrane (18, 19). In contrast, the protonated form (NH4^+^) diffuses much more slowly, with rates several orders of magnitude lower than ammonia. The addition of 2 mM NH_3_ to EdC revealed a shift in intracellular pH from 6.8 to 7.5 and an extracellular pH shift from 6.9 to 9.4 (Fig. S2A and C). Similar results were obtained by the addition of urea and its hydrolysis by urease activity (Fig. S2A and B).

### Ammonia and l-arginine could increase intracellular pH, but ammonia was more effective in boosting glycolysis and homolactic fermentation.

To identify other molecules able to provide the alkalization necessary to promote cell acidification, EdCs were treated with lactose and 2 mM l-Arg. l-Arg was a basic amino acid characterized by a guanidinium group, which has a pKa of 12.48 and was positively charged at physiological pH. The addition of 2 mM l-Arg resulted in an increase in extracellular pH from pH 6.9 to pH 9.4 and a modification of the pH_in_ up to 7.5, the same value was measured for urea/ammonia addition (pH_in_ 7.5) (Fig. S2B to D). However, the addition of 2 mM l-Arg was less effective in boosting acidification (Fig. S1B). Although a component of putative arginine ABC transport (arginine transport ATP-binding protein ArtM) was detected in the proteome (Table S1), we could not conclude that arginine was actively transported inside the cell through ArtM. However, increasing pH_in_ by exposing S. thermophilus cells to arginine confirmed that arginine was effective in alkalizing the intracellular environment. Conversely, no effect on pH_in_ was detected after the addition of NH_4_Cl (Fig. S2E). To further investigate the role of extracellular alkalization on the rate of glycolytic enzymes, EdCs were treated with lactose increasing the final NH_3_ or l-Arg concentration from 2 to 4 mM, and by a continue measuring of the extracellular pH by standard pH electrodes (Fig. S3). Even in these experimental conditions, although the maximum pH values reached after the addition of ammonia or arginine were comparable (pH 9.46 ± 0.06 for NH_3_ and pH 9.16 ± 0.06 for l-Arg), NH_3_ addition was more effective than l-Arg to boost acidification (Fig. S3), and l-Arg in the presence of lactose was more effective than lactose only. The differences between NH_3_ and l-Arg in triggering acidification have led to the hypothesis that NH_3_ may act on the activity of the enzymes involved in homolactic fermentation by alkalizing the cytoplasm because it was shown also by l-Arg and with other mechanisms.

Lactose, glucose, and l-lactic quantification confirmed what was deduced from the acidification plots (Table 1). The highest yield of homolactic fermentation (*yHlf*), which corresponded to the highest level of l-lactic acid produced, was measured after activation of EdC with lactose plus urea or ammonia. Interestingly, the latest conditions showed a low capacity of lactose intake (*cLacIn*), as shown by the residual lactose quantified after EdC activation (Table 1), compared to the high levels of *cLacIn* in all the other conditions tested. *cLacIn* showed an inverse correlation with the yield of glycolysis (*yGly*), which results in the extracellular accumulation of glucose (Table 1). In S. thermophilus, the mechanism of glucose export has not been described in detail, although glucose accumulation in the growth medium was already reported for some S. thermophilus mutants (20). To further investigate the role of extracellular alkalization on homolactic fermentation, lactose, glucose, and l-lactic have been quantified in EdCs blocked in transcription and translation and suspended in 100 mM Tris-HCl pH 8.0. The continuous monitoring of the extracellular pH showed that Tris-buffer maintained pH 8.0 ± 0.1 during the entire incubation time (Fig. S4). The highest values of *yHlf*, *cLacIn*, and *yGly* have been obtained with EdCs activated with lactose and ammonia (Table 1) followed by EdCs activated with lactose and l-Arg. These results led us to confirm that ammonia, was more effective than l-Arg in boosting the homolactic fermentation, irrespectively of media alkalization.

**TABLE 1:** Lactose, glucose, and lactic acid quantification in activated EdCs

Condition of activation of EdCs	Lactose (mM)	Glucose (mM)	Lactic acid (mM)	Homolactic fermentation yield (*yHlf)* (%)	Lactose intake capacity (*cLacIn*) (%)	Glycolytic yield (*yGly*) (%)
EdCs	ND	0.9 ± 0.9	0.3 ± 0.2			
EdCs, 14 mM lactose	1.6 ± 1.9	13.0 ± 1.0	0.5 ± 0.1	2.3	88	<0
EdCs, 14 mM lactose, 2 mM urea	8.6 ± 0.2	1.51 ± 0.08	6.5 ± 1.0	66.8	36.1	68.9
EdCs, 14 mM lactose, 2 mM urea, flurofamide 5 μM	1.0 ± 0.9	13.0 ± 1.0	0.6 ± 0.1	2.4	92.0	<0
EdCs, 14 mM lactose, 4 mM NH_3_	9.4 ± 0.4	1.5 ± 0.9	6.0 ± 1.2	74.2	30.1	63.6
EdCs, 14 mM lactose, 4 mM NH_3_, 45 mg/mL oxamate	3.6 ± 1.6	10.1 ± 0.9	0.8 ± 0.1	3.9	72.9	<0
EdCs, 14 mM lactose, 4 mM NH_4_Cl	10.3 ± 0.2	1.3 ± 0.1	2.2 ± 0.9	35.5	58.6	23.2
EdCs, 14 mM lactose, 4 mM l-arginine	1.4 ± 2.2	13.5 ± 0.9	0.8 ± 0.1	3.3	89.3	<0
EdCs, 14 mM lactose, pH 8.0	2.5 ± 0.7	10.3 ± 0.6	3.2 ± 0.1	13.8	82.4	10.6
EdCs, 14 mM lactose, 4 mM NH_3_, pH 8.0	1.0 ± 0.1	3.6 ± 0.2	18.9 ± 0.3	72.3	92.6	72.3
EdCs, 14 mM lactose, 4 mM l-arginine, pH 8.0	1.6 ± 0.1	9.0 ± 0.1	7.0 ± 0.7	28.0	88.9	27.7

In conclusion, acidification curves and intracellular pH measurements together with lactose consumption and lactic acid production in a pH-uncontrolled and a pH-controlled condition revealed that urea hydrolysis or ammonia or l-arginine addition was effective in determining an intracellular alkalization, but only urea hydrolysis or ammonia addition was able to strongly stimulate glycolysis and homolactic fermentation in EdCs. Therefore, based on previous observations and current data, it could be hypothesized that the role of ammonia may be 2-fold because it provides an intracellular alkalinization, which positively affects the activity of glycolytic enzymes, and it acts as a strong effector of enzymes involved in glycolysis and/or homolactic fermentation (7).

### Ammonia stimulated acidification in EdCs activated with lactose, glucose, and sucrose.

**FIG 3:**
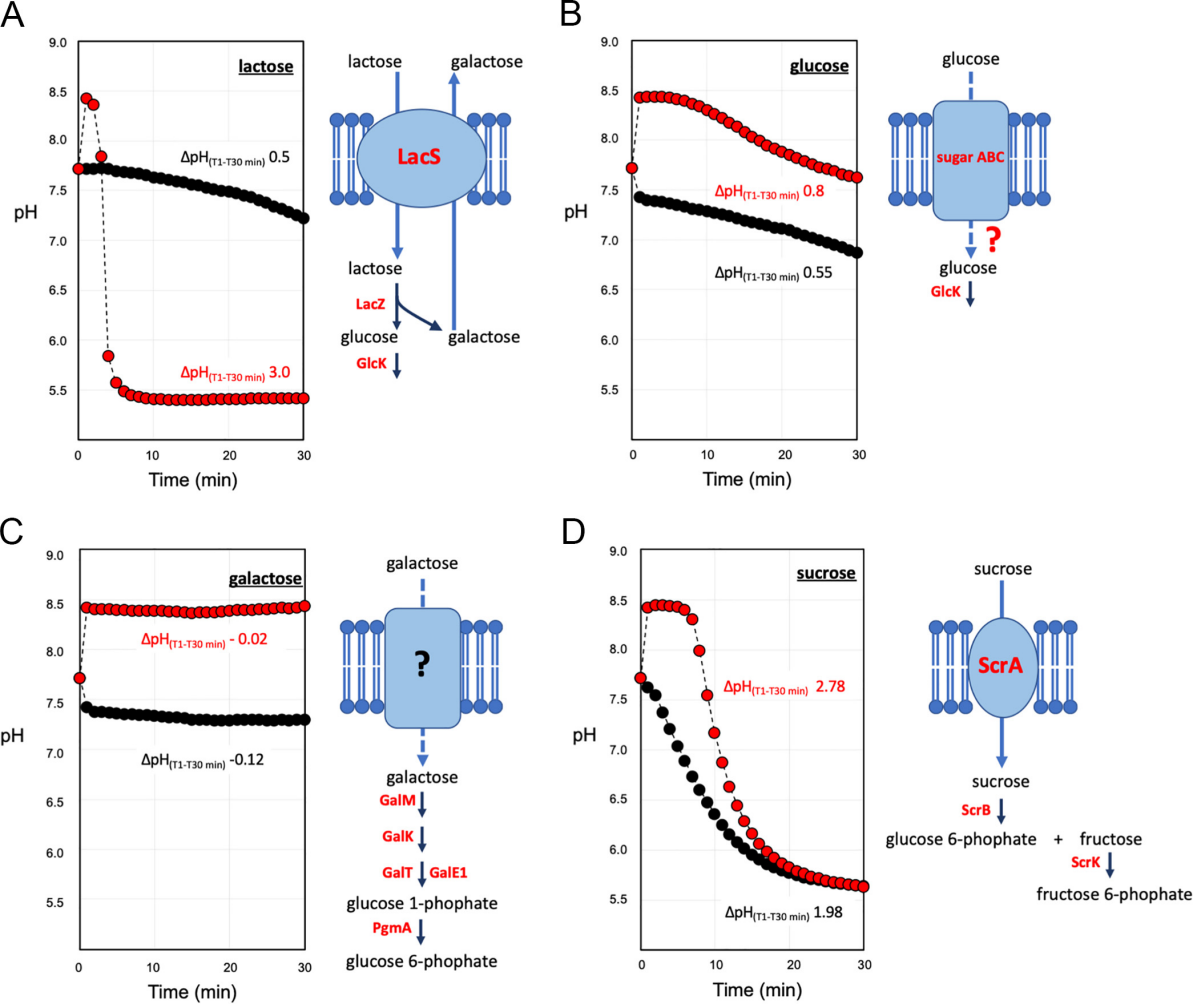
Activation of homolactic fermentation in EdCs with different carbon sources. (A) EdCs activated with lactose. (B) EdCs activated with glucose. (C) EdCs activated with galactose. (D) EdCs activated with sucrose. EdCs activated with sugar and NH_3_ (red circles). EdCs activated with sugar (black circles). ΔpH_(T1-T30 min)_ values are reported. Transport proteins and enzymes involved in sugar metabolism have also been reported. Transport proteins and enzymes detected in the proteome of EdCs are written in red. LacS, lactose permease. Sugar ABC, hypothetical glucose transporter. ScrA, sucrose PTS component II. LacZ, β-galactosidase. GlcK, glucose kinase. GalM, aldose 1-epimerase. GalK, galactokinase. GalT, galactose-1-phosphate uridylyltransferase. GalE1, UDP-glucose 4-epimerase. PgmA, phosphoglucomutase. Data are reported as the average of three replicates. The standard deviation was always below 2% of the measured pH.

Using carbon sources other than lactose, we confirmed the stimulating effect of ammonia on cells acidification (Fig. 3). The addition of glucose-induced acidification was comparable to that obtained using lactose as a carbon source (Fig. 3A and B). However, when glucose was supplemented with ammonia, the increase in the acidification rate was not comparable to that observed with lactose (Fig. 3B). Glucose is a non-phosphotransferase system (PTS) sugar and is considered a poor substrate for the growth of some S. thermophilus strains (4). Although specific glucose transport has not been identified in S. thermophilus (2, 4), proteomic data revealed the presence of an ABC transporter sugar permease (Table S1) that could be involved in glucose transport. Galactose was ineffective in stimulating acidification in EdCs (Fig. 3C). S. thermophilus MIM13 is a galactose-negative strain despite genes coding for the Leloir pathway being maintained in their genomes (9, 21). Proteomic data revealed that the enzymes of the Leloir pathway were all present in EdC, specifically galactokinase (GalK), galactose-1-phosphate uridylyltransferase (GalT), galactose-1-epimerase (GalM), and uridine diphosphate (UDP)-glucose 4-epimerase (GalE1) (Fig. 3C and Table S1). Therefore, the inability of EdC to activate homolactic fermentation in the presence of galactose could be due to the absence of a galactose transport system. More interestingly, when sucrose was used as a carbon source, a strong acidification rate was observed with a ΔpH_(T1-T30 min)_ of 1.98, which increased to 2.78 when ammonia was supplemented (Fig. 3D). The prompt activation of fermentation by sucrose underlined that the genes coding for sucrose transport and hydrolysis (*scrR*, *scrB*, *scrA,* and *scrK*) were induced even if EdC had been prepared by growing S. thermophilus MIM13 on lactose as a carbon source. Proteomic data confirmed the presence of all proteins required to catabolize sucrose, specifically PTS beta-glucoside transporter (ScrA), sucrose-6-phosphate hydrolase (ScrB), fructokinase (ScrK), and sucrose operon repressor (ScrR) (Fig. 3D and Table S1).

Considering that glucose and fructose enter the glycolytic pathway in S. thermophilus, the differences in the acidification curves reported in Fig. 3 for lactose, glucose, and sucrose could be related to differences in the capacity of sugar uptake across the cell membrane, with lactose > sucrose > glucose. Moreover, the capacity of sugar transport does not seem to be related to the type of membrane transport because glucose and lactose are non-PTS sugars, whereas sucrose transport is governed by a phosphoenolpyruvate-phosphotransferase system (PEP-PTS) (2, 4).

### NH_4_^+^ was a strong effector of S. thermophilus phosphofructokinase.

One of the key enzymes known to exert a regulatory role on the glycolytic pathway is phosphofructokinase (PFK) (22). We, therefore, considered whether ammonia could stimulate the activity of the PFK of S. thermophilus. PFK activity, quantified on total protein extracts of EdC, revealed that the addition of 0.5 mM NH_3_ to the assay mix solution did not affect the pH of the PFK assay buffer, which maintained the value of pH 7.4 ± 0.2. The data reported in Table 2 showed that 0.5 mM ammonia resulted in a 122% increase in PFK activity and that a 304% increase in PFK activity was achieved in the presence of 0.5 mM NH_4_Cl. Considering that at a physiological pH of 7 to 7.5, ammonia (NH_3_) and ammonium (NH_4_^+^) rapidly reach equilibrium, with NH4^+^ being the dominant form, we assume that the molecular species that acts as an effector of PFK was NH_4_^+^. The addition of 0.5 mM ammonia or 0.5 mM NH_4_Cl to a purified PFK enzyme of *Bacillus* sp. (the positive-control provided in the PFK assay kit) resulted in 281% and 591% increases in PFK activity, respectively, thereby confirming the role of NH_4_^+^ as an effector of PFK in *Bacillus* sp. (Fig. S5).

**TABLE 2:** PFK activity

Sample	PFK activity^*a*^ (nmol/min/mL)	% Activity increment using NH_3_ or NH_4_^+^ as PFK effectors
S. thermophilus EdC total protein extract	575 ± 166	NA
S. thermophilus EdC total protein extract, 0.5 mM NH_3_	1278 ± 193	122
S. thermophilus EdC total protein extract, 0.5 mM NH_4_Cl	2321 ± 332	304
*Bacillus* sp. PFK	8.4 ± 0.7	NA
*Bacillus* sp. PFK, 0.5 mM NH_3_	32 ± 9	281
*Bacillus* sp. PFK, 0.5 mM NH_4_Cl	58 ± 14	591

a PFK activity is reported as nmol/min/mL = milliunit/mL. One unit of PFK is the amount of enzyme that will generate 1.0 μmol of NADH per minute at pH 7.4 at 37°C. NA, not applicable.

## DISCUSSION

S. thermophilus is considered a unicity among lactic acid bacteria due to urease activity, which represents a distinctive character of the species. Urease is a complex enzyme whose subunits and accessory proteins are encoded by 11 genes that account for approximately 0.9% of the core genome of the species (23). S. thermophilus urease activity has been extensively studied because it exerts a delayed effect on milk acidification (24–26). Urease activity was reported to be correlated with aspartate, glutamine, and arginine biosynthesis and carbon dioxide metabolism (10–13). Moreover, we previously showed that urea hydrolysis increases the catabolic yield of S. thermophilus by modulating the intracellular pH and increasing the activity of β-galactosidase, glycolytic enzymes, and lactate dehydrogenase (7).

Urea hydrolysis results in increases in both the intracellular and extracellular pH due to the release of ammonia and its rapid diffusion outside the cell. Urease activity is not considered essential for S. thermophilus, and urease-negative mutants can still grow efficiently in milk. However, urease deficiency results in a decreased rate of acidification and bacterial counts in milk (15, 16). More recently, it was reported that the urease activity of S. thermophilus showed a significant correlation with the use of lactose and the production of lactic acid and acetaldehyde (26). We could, therefore, summarize that the positive effect of urease on S. thermophilus metabolism is the result of several factors, including the modulation of the intracellular and extracellular pH, CO_2_, and NH_3_ supply to anabolic pathways. However, it is difficult to consider urease activity as an acid stress response, which was reported for Streptococcus salivarius and Helicobacter pylori, because in S. thermophilus, urease biosynthesis is induced before the environmental pH values could be considered critical for its growth (pH close to 6) (25, 27, 28). Here, we showed that half of the urea supplemented in milk was indeed hydrolyzed after 90 min of incubation when the pH was 6.1 (Fig. 1C). Urease activity is, therefore, induced before the environmental pH becomes critical to prevent the stress and not as a response to acidic stress. This anticipation will provide a double benefit: a delay of the acidification and a boost of glycolysis and homolactic fermentation, allowing the microorganism to grow faster and thereby having a fitness advantage.

Another point of debate is related to the role of NH_3,_ generated by urea hydrolysis, on the acidification rates and bacterial counts of S. thermophilus. Ammonia was reported to be effective in the modulation of intracellular pH and in boosting homolactic fermentation (7, 29). More recently, it was reported that milk supplemented with (NH_4_)_2_SO_4_ accelerates the acidification process, but this effect was observed in only one S. thermophilus strain analyzed (14). In this context, it should be emphasized that both ammonia and ammonium salts can be used as nitrogen sources by S. thermophilus. However, while ammonium ions require specific membrane transport, ammonia can freely diffuse across the cell membrane. The results obtained in this study strongly support the hypothesis that ammonia and ammonium ions can boost the homolactic fermentation of S. thermophilus.

Previous studies reported that in S. thermophilus, the modulation of the intracellular pH toward alkaline values, controlled by urea hydrolysis, did not affect the transcription of the genes coding for lactose permease (*lacS*), β-galactosidase (*lacZ*), glyceraldehyde-3-phosphate dehydrogenase (*gapA1*), phosphoglycerate kinase (*pgk*), pyruvate kinase (*pyk*) and lactate dehydrogenase (*ldh*) (7). We, therefore, decided to test the effect of urease activity, ammonia, and ammonium salt on homolactic fermentation of nongrowing cells of S. thermophilus blocked in transcription and translation by rifamycin and chloramphenicol treatment. The results obtained highlight that urea, specifically ammonia generated by urea hydrolysis or supplemented with ammonium hydroxide, was particularly effective in promoting homolactic fermentation in EdCs. Under all the other experimental conditions, where the cell suspension was supplemented with lactose or lactose and urea and flurofamide (Fig. 2B) or with ammonium chloride (Fig. S1), the acidification was less pronounced. It was, therefore, assumed that ammonia increased homolactic fermentation and the glycolysis yield, whereas it reduced the lactose intake. In contrast, in the absence of glycolytic activation, the capacity of lactose intake strongly increased. This led to the hypothesis that glycolytic intermediates could strongly act on the regulation of the lactose permease LacS. In S. thermophilus, LacS activity is positively modulated by phosphorylation mediated by HPr(His~P), a component of the PEP-PTS system. However, HPr, which can phosphorylate LacS only if it is HPr(His~P), can also be phosphorylated on a serine residue (HPr[Ser-P]) by a cytosolic HPr(Ser) kinase that in several Gram-positive bacteria is stimulated by fructose 1,6-biphosphate (FBP), an early glycolytic intermediate, but no such activation was reported for S. thermophilus (3, 30–33). In contrast, it was reported that serine phosphorylation of HPr in cell extracts of S. thermophilus was stimulated upon the addition of ATP, and HPr(Ser-P) dephosphorylation was triggered by P_i_ (3). Based on the above considerations, we propose that in energetically discharged cells (EdCs), the low level of ATP and the consequent high level of P_i_ will decrease the concentration of HPr(Ser-P), favoring an increase in the HPr(His~P)/HPr(Ser-P) ratio with a positive effect on LacS phosphorylation and lactose intake (Fig. S5) (7). Interestingly, when EdCs were prepared in a solution buffered at pH 8.0, the lactose intake (*cLacIn*) reached a high value also when lactose was supplemented with NH_3_ (Table 1), thus indicating that the transport system and/or the β-galactosidase, which cooperate with the antiporter LacS by providing galactose, are highly active at alkaline pH, as previously observed (3, 7).

As shown in Fig. 2 and 3 and Fig. S1, the starting pH_in_ of EdCs was not simple to practically control, despite EdCs having been prepared with a highly standardized protocol. Likewise, the rate of acidification of activated EdCs was not the same in the different lots of EdC. However, these differences between batches of EdCs have never been relevant in questioning the effect of ammonia in stimulating glycolysis and lactic fermentation, and when the experiments were carried out in a solution buffered at pH 8, the role of ammonia as a positive effector of glycolytic enzymes was further confirmed. At pH 8 the values of *yHlf* and *yGly* were, respectively, about 6 and 7 times higher than those calculated for the EdCs activated with lactose, and about three times higher than those calculated for the EdCs activated with lactose and arginine. By comparing lactose and lactose plus arginine conditions, the higher values of *yHlf* and *yGly* obtained with arginine supplementation could be related to the intracellular alkalization which is determined by arginine (Fig. S2D).

**FIG 4:**
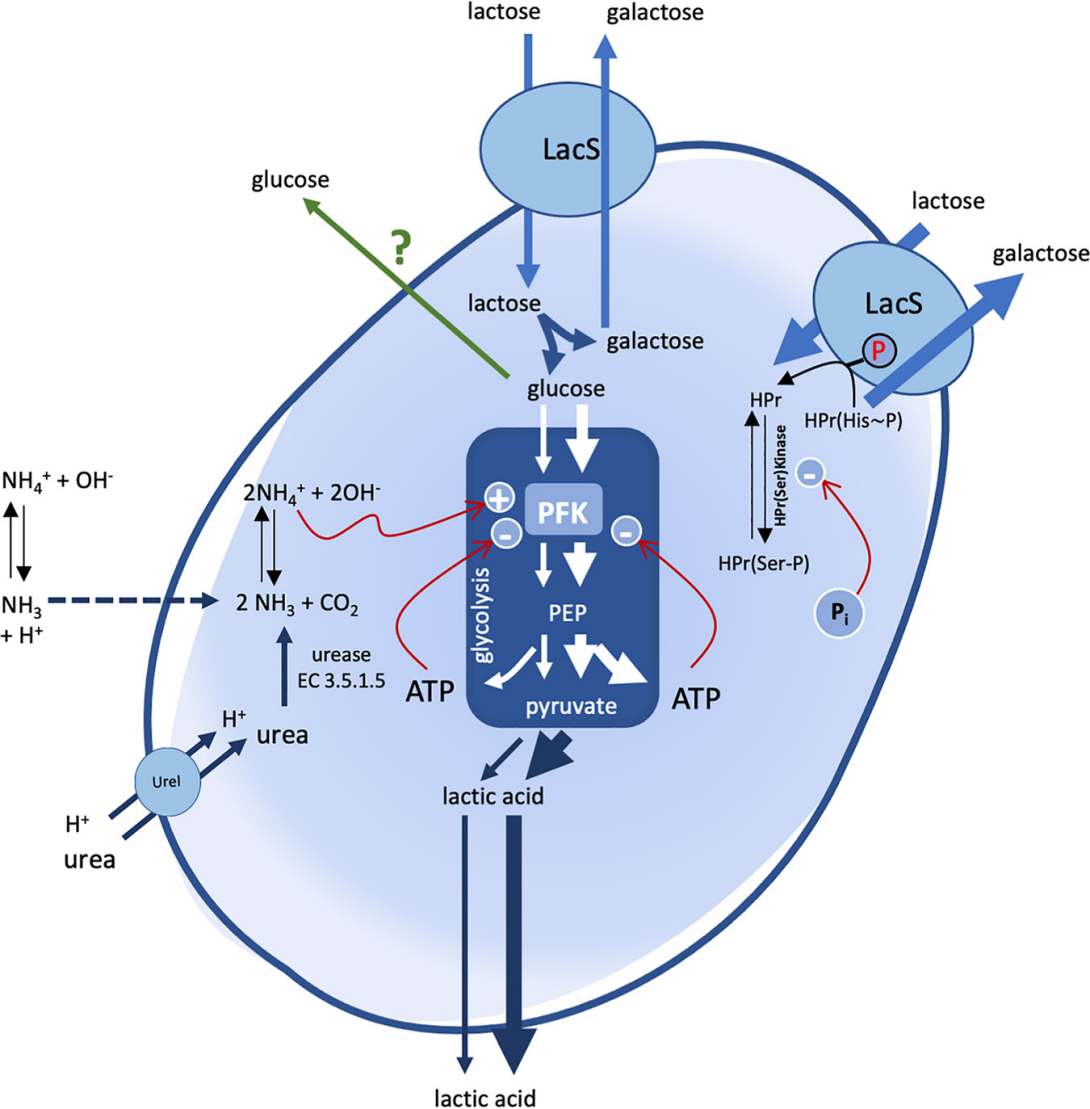
Metabolic scheme describing the lactose homolactic fermentation of S. thermophilus in the presence of urea and ammonia. Glycolysis is represented by the blue box in the center of the cell. Arrow thickness is proportional to the capacity of sugar transport, glycolytic flux, or homolactic fermentation. The dashed arrow refers to the free diffusion of NH_3_ across the cell membrane. The green arrow refers to the exit of glucose outside the cells, the mechanism of which is still unknown. Red arrows refer to positive (+) or negative (−) allosteric effectors of phosphofructokinase (PFK) and HPr(Ser)Kinase. LacS, lactose permease. UreI, urea transport permease. PEP, phosphoenolpyruvate.

The overall data collected in this study highlighted that cells of S. thermophilus benefit from the ammonia generated by urea hydrolysis or supplied as ammonium hydroxide to boost catabolism (Fig. 4). It follows the paradox that in S. thermophilus, to improve acidification by homolactic fermentation, it is first necessary to provide alkalization. However, intracellular pH measurement and supplementation of l-arginine together with the quantification of the homolactic fermentation yield strongly suggest that ammonia addition to a cell suspension stimulates homolactic fermentation by acting on the third layer of the cellular regulatory mechanism, which exclusively modulates the kinetic parameters of the enzymes. Ammonia can freely pass across the cell membrane, whereas NH_4_^+^ diffuses much more slowly (18, 19). In this context, and based on previous observations, we hypothesize that the intracellular alkalization by ammonia generated by urea hydrolysis or added to the medium could be responsible for the boost of homolactic fermentation by increasing the activity of the enzymes involved in this catabolism, which display an optimum activity at alkaline pH (7, 29). Once inside the cell, ammonia goes in equilibrium with its protonated form NH_4_^+^. Therefore, both NH_3_ and NH_4_^+^ could act as activators of glycolytic enzymes. In Lactococcus lactis, it has been shown that phosphofructokinase (PFK) exerts strong control over the glycolytic flux, and the increase in PFK activity results in a proportional increase in specific rates of glucose uptake and lactate formation (34–36). In Escherichia coli, it has been reported that NH_4_^+^ enhances glucose uptake under aerobic conditions, whereas in the baker’s yeast Saccharomyces cerevisiae, NH_4_^+^ is known to cause a general stimulation of PFK, abolishing its sensitivity to ATP (37, 38). Moreover, it has been reported that the AMP deaminase reaction as an ammonia-forming reaction can participate in the stimulation of PFK or glycolytic flux in S. cerevisiae (39). The role of ammonia in the cell bioenergetics of S. thermophilus is not new. The first evidence emerged by studying the physiological role of S. thermophilus urease, and the effect of urease on the protocooperation between S. thermophilus and Lactobacillus delbrueckii subsp. *bulgaricus* in milk (7, 29). Here, we showed that, in S. thermophilus, ammonium acts as a strong effector of PFK (Table 2), thus supporting the hypothesis that the intracellular or extracellular supply of ammonia stimulates glycolysis and, in cascade, homolactic fermentation.

In conclusion, in S. thermophilus, the effect of urea hydrolysis on energetic metabolism is related to the generation of ammonia and the effect of the latter as a modulator of the glycolytic enzyme PFK (as ammonium ions). This is the first evidence that shows the stimulating effect on glycolysis and homolactic fermentation of ammonia directly supplied to a cell suspension and a culture medium during bacterial growth. Enzymatic assays revealed that ammonium is more effective than ammonia as a PFK effector. These data are not in contradiction to what was observed in intact live S. thermophilus cells where ammonium supplementation was not as effective as ammonia in the stimulation of homolactic fermentation. Ammonia acts as a “friendly” Trojan horse by freely passing across the cell membrane, and once inside going in equilibrium with its protonated form NH_4_^+^, it can exert its role as a strong PFK effector (Fig. 4).

Due to the high level of conservation of the glycolytic pathway among living organisms, we speculate that all ammonia-generating pathways could boost the glycolytic flux acting on PFK activity, which was demonstrated for AMP deaminase of S. cerevisiae (39). We, therefore, propose to reevaluate the physiological role of ammonia-generating reactions in other organisms, considering their possible role in the modulation of energetic metabolism.

## MATERIALS AND METHODS

### Monitoring of S. thermophilus growth and metabolism in milk.

S. thermophilus MIM13 grown in M17 (20 g/liter, lactose) was collected in the late exponential growth phase (optical density at 600 nm [OD_600_] of 2.5), washed, quantified by flow cytometry (FCM) (17), and used to inoculate 100 mL of sterile skimmed milk (110°C for 15 min) preheated at 37°C supplemented with ^13^C-urea (4 mM) (Merck, Italy), with and without the addition of the urease inhibitor flurofamide (5 μM) (8). Bacterial growth was monitored by FCM using a BD Accuri C6 Plus (Becton, Dickinson, Italy), and milk acidification was monitored by a standard pH meter. For total cell quantification by flow cytometry, 500 μL of milk culture was subjected to a clearing procedure by adding an equal volume of Tris-HCl (2 M) EDTA (0.2 M) buffer (pH 8) (15). Following 10 min of incubation at 50°C, the cell suspension was labeled with SYBR Green I (Sigma-Aldrich, Milan, Italy). After incubation at 37°C for 15 min, the labeled cell suspension was diluted to reach approximately 10^6^ events per mL and analyzed by FCM. The obtained data were analyzed using BD Accuri C6 Plus software version 1.0 (BD Biosciences, Milan, Italy). The SYBR green I fluorescence intensity of stained cells was recovered in the FL1 channel. Density plots of green fluorescence (FL1) versus FSC allowed for an optimal distinction between the SYBR green I-stained microbial cells and instrument noise or sample background. An electronic gate on the green fluorescence/FSC density plot was used to select and measure the total bacterial concentration, which was reported as fluorescent units per mL (FU/mL).

For ^13^C-urea and lactose consumption and lactic acid production, samples were taken at defined time intervals and immediately stored at −80°C. ^13^C-urea, lactose, and lactic acid quantification were carried out by NMR. The ^13^C NMR spectra were recorded on a Bruker AV600 spectrometer operating at a frequency of 150.93 MHz, equipped with a 5 mm TXI inverse probe and z-axis gradients at 25°C. NMR experiments were performed using a 5-mm NMR tube containing 600 μL of solution. All ^13^C NMR spectra were referenced to an external solution of 5 M sodium formate (^13^C 99%) in D_2_O set to 172 ppm. The deuterium in the capillary was enough for the lock system without changes in the concentration of the considered samples. The ^13^C NMR acquisition parameters for all samples were as follows: 10 μs acquisition pulse 60°, 14 s relaxation delay, 37879 Hz spectral width; 0.9 s acquisition time, 400 scans, 64 K time domain. Proton broad-band decoupling was achieved by a Waltz-type pulse sequence. Chemical shifts (δ) were measured in ppm. The NMR spectra were transformed with line broadening (LB = 6.0 Hz and GB = 0.02) by TOPSPIN software, and the baseline was corrected using a polynomial function.

Standard solutions of different concentrations of lactose (30 to 50 g/liter), urea (0.5 to 4 mM g/liter), and lactic acid (0.4 to 3 g/liter) were prepared for the quantitative measurements. Selected signals were deconvoluted by Lorentzian lineshape to calculate their areas. In detail, the signals lying at 104 ppm and 97 ppm for the lactose solutions (anomeric carbons) and lying at 164 ppm (CO) and 21.7 ppm (CH3) for the urea and lactic acid solutions, respectively, were considered. Different calibration lines were obtained by plotting the ratio of the areas of these ^13^C signals of standard solutions and the area of the reference signal versus the concentration expressed in g/liter. The complete assignment of all carbon signals was performed according to Lu et al. (40).

### Effect of urea and ammonia on homolactic fermentation in nongrowing S. thermophilus.

To discriminate between the two hypothesized roles of urea-derived ammonia, a nitrogen source or an alkalizing agent, the triggering effect of urease activity and ammonia on homolactic fermentation of S. thermophilus MIM13 was monitored on nongrowing cells blocked in transcription and translation machinery by 20 mg/mL rifamycin and 100 mg/mL chloramphenicol (41, 42). To this aim, S. thermophilus cells grown in M17 (20 g/liter, lactose) were collected in the late exponential growth phase (OD_600_ = 2.5), washed, and suspended in solution A (9 g/liter NaCl, 100 μg/mL chloramphenicol, 20 μg/mL rifamycin). Cells treated with 20 mg/mL rifamycin and 100 mg/mL chloramphenicol were not able to produce colonies when plated on M17 agar plates containing the same concentration of the two antibiotics. According to previous data, the cell suspension was de-energized through incubation at 37°C in the presence of 4 mM urea (7). After incubation, the cell suspension was washed several times with solution A to remove the excess ammonia until reaching a stable pH. Cells prepared as described above, concentrated to reach a final density of 10^9^ FU/mL, were called energetically discharged cells (EdCs) (7). EdCs were preheated at 37°C, supplemented with 0.8 μM pH-sensitive 6-carboxyfluorescein, and dispensed (200 μL) in 96-well microtiter plates. When requested, cell metabolism was activated by adding 14 mM lactose, sucrose, fructose, or galactose with and without the addition of 4 mM urea, 2 mM NH_3_, 8 mM NH_4_Cl, or 100 μM flurofamide. The pH-dependent carboxyfluorescein fluorescence was recorded every 1 min for 30 min using a Victor 3 fluorometer (Perkin-Elmer, Waltham, MA). An aliquot of EdCs supplemented with 0,8 μM 6-carboxyfluorescein (EdCs) was used for pH *versus* fluorescence calibration. To this aim, the pH of EdCs supplemented with 100 μM gramicidin to equilibrate the intracellular pH with the extracellular pH of 5 was set to values ranging from 4.69 to 9.78 using ammonia and lactic acid, the pH was measured using a standard pH meter, and the fluorescence intensity was measured using a Victor 3 fluorometer. When requested, EdCs or EdCs prepared in 100 mM Tris-HCl pH 8.0, preheated at 37°C have been dispensed (15 mL) in 50 mL tubes, activated by adding 14 mM lactose with and without the addition of 2 to 4 mM NH_3_ or l-Arg and the pH continuously monitored by standard electrodes using an iCINAC apparatus wired version (KPM Analytics, Guidonia, Italy).

Lactose, glucose, and l-lactic acid have been quantified enzymatically using the UV method for the determination of foodstuffs and other materials (R-Biopharm Italia Srl, Melegnano MI, Italy). The homolactic fermentation yield (*yHlf*) was calculated as the % of l-lactic acid produced on the l-lactic acid expected to be synthesized based on the amount of lactose consumed considering that only the glucose moiety of lactose is metabolized in S. thermophilus $\mathrm{yHlf}=L-\text{lactic acid }(\text{mM})/\left[\text{lactose consumed }\left(\text{mM}\right)\times 2\right]\times 100$. The glycolytic yield (*yGly*) was calculated as the % of glucose catabolized on the glucose expected to be catabolized based on the amount of lactose consumed $\mathrm{yGly}=100-(\text{glucose }\left(\text{mM}\right)/\text{lactose consumed }\left(\text{mM}\right)\times 100)$. The lactose intake capacity (*cLacIn*) was calculated as the % of lactose consumed on the total lactose provided to the EdCs (lactose t0) $\mathrm{cLacIn}=\text{(lactose consumed }\left(\text{mM}\right)/\text{lactose t}0\;\left(\text{mM}\right))\times 100$. The lactose consumed was calculated as the difference between the lactose provided to EdCs (lactose t0) and the residual lactose after 30 min of incubation, lactose t30, lactose consumed (mM)=(lactose t0-lactose t30).

### Measurement of intracellular pH.

The intracellular pH (pH_in_) of S. thermophilus EdCs was measured using the pH-sensitive fluorescence probe 5 (and 6-)-carboxyfluorescein succinimidyl ester (cFSE) (29). EdCs were obtained as described above, diluted to an OD_600_ of approximately 0.5, and supplemented with 4 μM cFDASE (Sigma-Aldrich, Milan, Italy), which is a precursor molecule of cFSE. The suspensions were then incubated for 30 min at 37°C. During this incubation, the membrane-permeating cFDASE was cleaved by intracellular esterases, and the resultant cFSE molecules were conjugated to the aliphatic amines of intracellular proteins. After centrifugation at 15,000 × *g* for 1 min and washing with solution A, the cells were suspended in the same volume of solution A. The unconjugated probe was eliminated by the addition of glucose at a final concentration of 16 mM and subsequent incubation at 37°C for 1 h. The cFSE fluorescence intensity of stained cells was recovered in the FL1 channel (excitation 488 nm, emission filter 530/30, provided by BD Biosciences, Milan, Italy). Density plots of green fluorescence (FL1) and FSC allowed for an optimal distinction between the cFSE-stained microbial cells and instrument noise or sample background. An electronic gate on the green fluorescence/FSC density plot was used to select the measured bacterial concentration (events per mL), and the selected data from the bacterial gate were subsequently visualized on a cFSE green fluorescence histogram for further analysis. To measure the pH_in_, 5 mL of a cFSE-labeled cell suspension was incubated at 42°C in the presence or absence of 2.5 mM urea, 2 mM ammonia, 2 mM ammonium sulfate, or 2 mM l-Arg for 5 min. The pH_in_ was determined by measuring the fluorescence intensities in the FL1 channel via flow cytometry before and after 5 min of incubation at 37°C. A calibration curve that reported FL1 fluorescence versus pH_in_ was obtained as described below. An aliquot of the cell suspension was washed and suspended in different buffers with pH values ranging from 5.62 to 7.47 and treated with 100 μM gramicidin (Sigma-Aldrich, Milan, Italy), which dissipates the transmembrane proton gradient. The fluorescence intensity was then measured for calibration at appropriate external pH values. (Fig. S2).

### Proteomic analysis of EdCs.

Proteomic analysis of EdCs was performed at UNITECH OMICs (University of Milan, Italy). To this aim, EdCs were collected and suspended in 0.1 M Tris-HCl buffer pH 8 and subjected to mechanical lysis using a Precellys 24 Tissue Homogenizer (Bertin Instruments, Montigny-le-Bretonneux, France). After the reduction in 5 mM DTT at 52°C for 30 min, the sample was subjected to alkylation in 15 mM IAA for 30 min in the dark. Trypsin digestion was performed at 37°C, pH 8, for 18 h using 1 μg of trypsin (Merck, Milan, Italy). The reaction was stopped by adding 50% TFA. The sample was then analyzed using a Dionex Ultimate 3000 nano-LC system (Sunnyvale CA, USA) connected to an Orbitrap FusionTM TribridTM mass spectrometer (Thermo Scientific, Bremen, Germany) equipped with a nanoelectrospray ion source. Peptide mixtures were preconcentrated onto an Acclaim PepMap 100 − 100 μm × 2 cm C18 (Thermo Scientific) and separated on an EASY-Spray ES802 column, 25 cm × 75 μm ID packed with Thermo Scientific Acclaim PepMap RSLC C18, 3 μm, 100 Å. The temperature was set to 35°C. The peptides were eluted with gradient: from 96% buffer A (0.1% formic acid in water) to 40% buffer B (0.1% formic acid in water/acetonitrile [2/8]). Total gradient: 110 min. Constant flow rate: 300 nL/min. Total run: 144 min. MS spectra were collected over an *m/z* range of 375 to 1500 Da at 120,000 resolutions, operating in the data-dependent mode, with a cycle time of 3 sec between master scans. HCD was performed with collision energy set at 35 eV. Polarity: positive. Data were analyzed using Proteome Discoverer 2.2 software by setting the uniprot-Streptococcus Thermophilus fasta and trypsin databases as digestion enzymes.

### PFK assay.

PFK activity was measured on the total protein extract of EdCs using the Phosphofructokinase (PFK) Activity Colorimetric assay kit (Sigma-Aldrich, Milan, Italy). To this aim, EdCs were prepared as described previously and subsequently subjected to total protein extraction. Mechanical cell disruption was carried out using a Precellys bead beater (Advanced Biotech Italia SRL, Seveso, Italy). To minimize the interference of NADH-generating enzymatic activities, the PFK assay was performed in the presence of 30 nM Br-Acivicin, a chemical inhibitor of glyceraldehyde-3-phosphate dehydrogenase (43). Several sample dilutions were tested to ensure that the readings were within the linear range of the standard curve. After this setup, S. thermophilus EdCs total protein extract diluted 1 to 200 in PFK buffer was used in the reaction. When necessary, NH_3_ or NH_4_Cl was added to the reaction mix to reach a final reaction concentration of 0.5 mM. The addition of 0.5 mM NH_3_ to the PFK buffer did not interfere with the PFK buffer pH.

### Data availability.

The mass spectrometry proteomics data have been deposited to the ProteomeXchange Consortium via the PRIDE partner repository with the data set identifier PXD031867 (44).
