# Supplementary material for: In Streptococcus thermophilus, Ammonia from Urea Hydrolysis Paradoxically Boosts Acidification and Reveals a New Regulatory Mechanism of Glycolysis
Source: Microbiol Spectr. 2022 Apr 25;10(3):e02760-21. doi: 10.1128/spectrum.02760-21 (PMC9241937; doi:10.1128/spectrum.02760-21)
Supplement: SUPPLEMENTAL FILE 2 — Supplemental material. Download spectrum.02760-21-s002.pdf, PDF file, 0.7 MB [file spectrum.02760-21-s002.pdf]

Supplementary Fig. 1

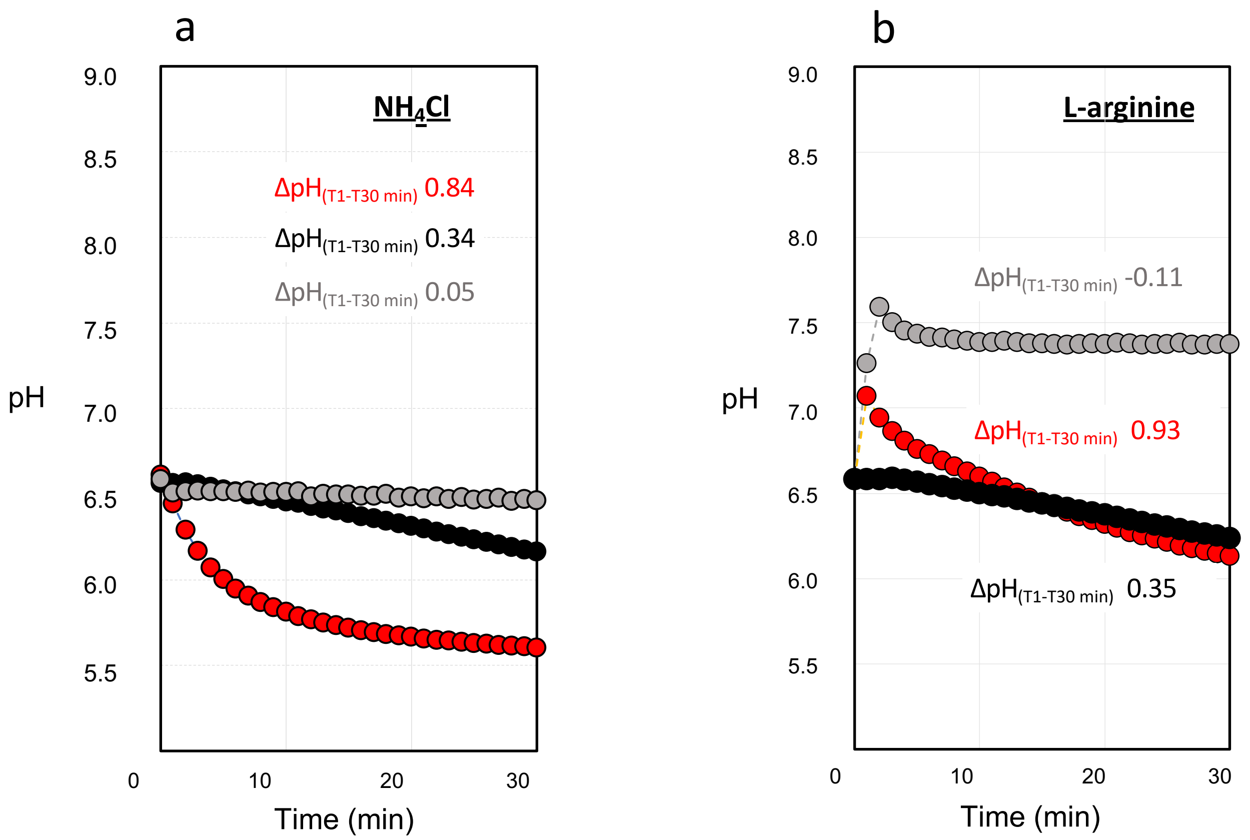

Activation of homolactic fermentation in EdCs with 14 mM lactose supplemented with 2 mM ammonium chloride (a) or 2 mM L-Arg (b). Red circles, EdCs activated with lactose and 2 mM ammonium chloride or L-Arg. Black circles, EdCs activated with lactose. Grey circles, EdCs activated with ammonium chloride or L-Arg. $\Delta pH_{(T1\text{-}T30\ min)}$ values are reported. Data are reported as the average of three replicates. The standard deviation was always below 2% of the measured pH.

Supplementary Fig. 2

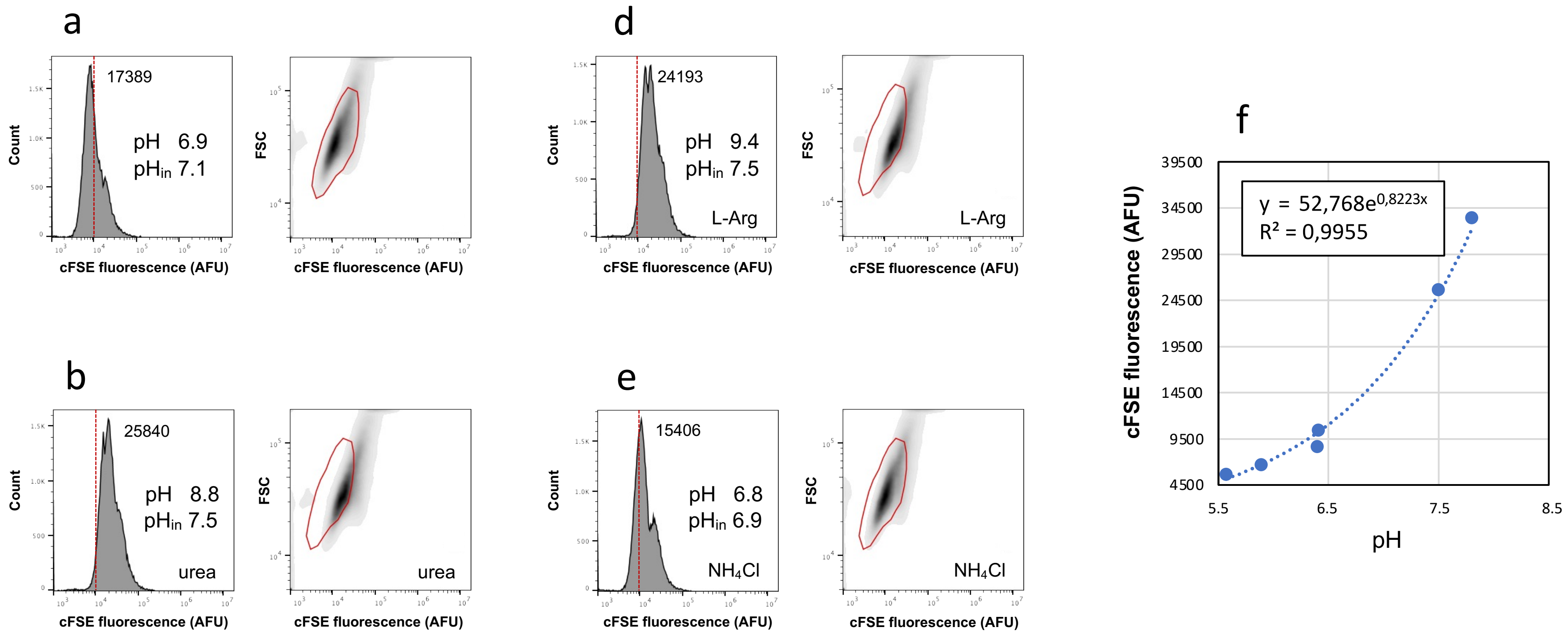

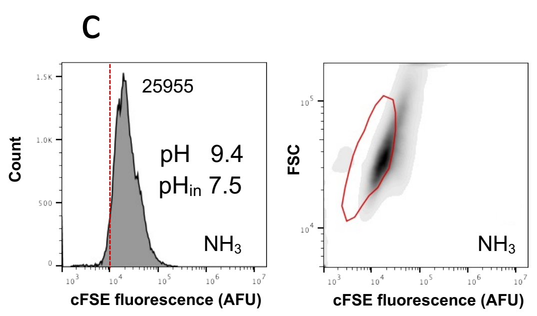

The effect of urea, ammonia, L-Arg, and ammonium chloride on variations in the pH_{in} and medium pH of the EdCs of *S. thermophilus* MIM13. Intracellular pH shift is represented by frequency histogram and density plots showing cell count and cells FSC *vs.* cFSE fluorescence after 5 min of incubation at 37 °C. (a) EdCs. (b) EdCs supplemented with 5 mM urea. (c) EdCs supplemented with 2 mM NH_3. (d) EdCs supplemented with 2 mM L-Arg. (e) EdCs supplemented with 2 mM NH_4Cl.
The red vertical lines refers to the average cFSE fluorescence of EdCs. The red gate and the red vertical lines are used to appreciate the fluorescence shift generated by intracellular alkalization. pH_{in} was measured based on the calibration curve in (F).

Supplementary Fig. 3

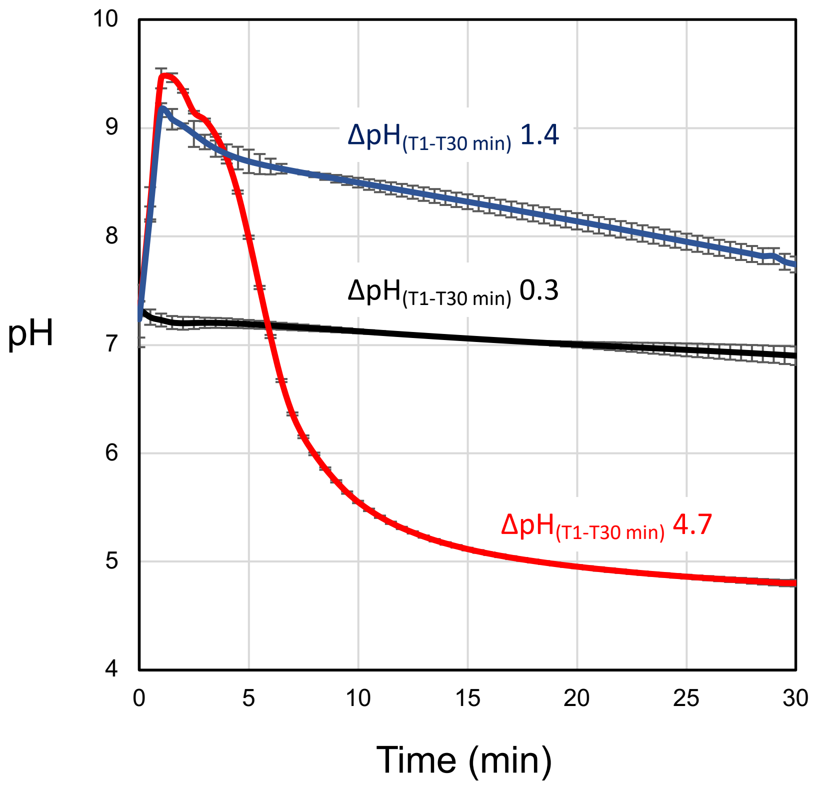

max pH reached

EdCs-lactose-NH_3, pH 9.46 ± 0.06

EdCs-lactose-L-Arg, pH 9.16 ± 0.06

Activation of homolactic fermentation in EdCs with 14 mM lactose (black line) supplemented with 4 mM ammonia (red line) or 4 mM L-Arg (blu line). $\Delta pH_{(T1\text{-}T30\ min)}$ values are reported. Data are reported as the average of two replicates. Error bars represent the standard deviation. The max pH value reached in samples supplemented with ammonia or L-Arg are shown in figure.

Supplementary Fig. 4

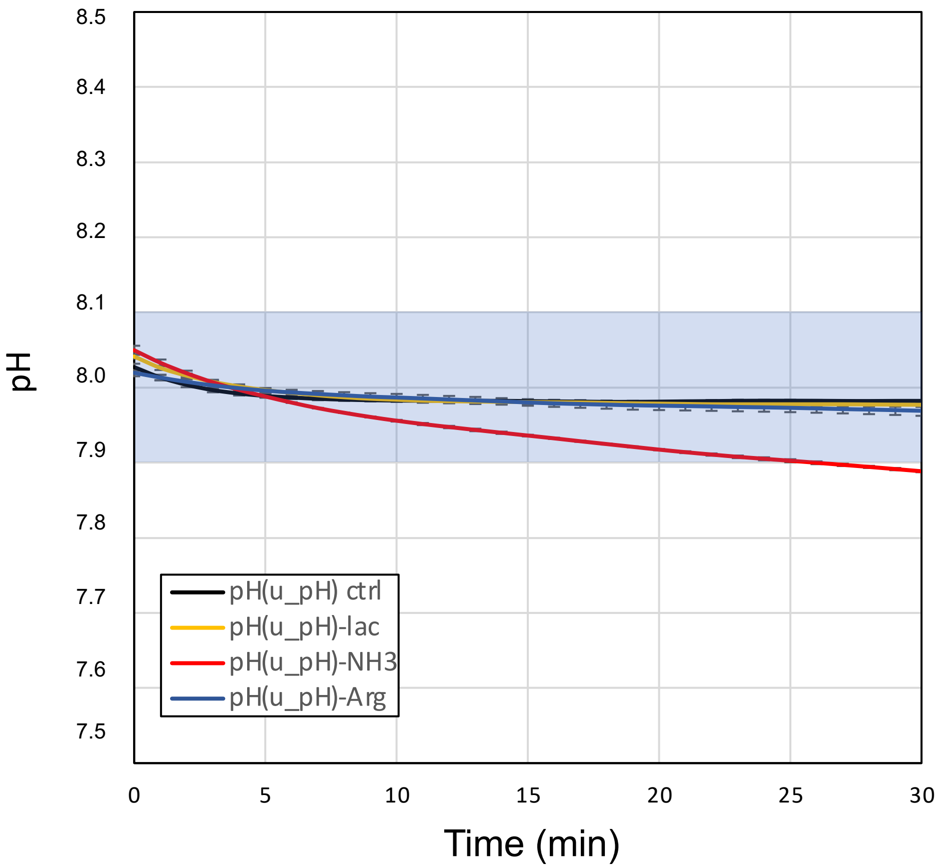

Monitoring of extracellular pH during activation of EdCs suspended in 100 mM TRIS-HCl pH 8.0. Black line, EdCs. Yellow line, EdCs activated with 14 mM lactose. Red line, EdCs activated with 14 mM lactose and 2 mM NH_3. Blu line, EdCs activated with 14 mM lactose and 2 mM L-Arg. Incubation temperature, 37 °C. pH was recorded every min using iCINAC apparatus. Error bars represent the standard deviation calculated on two replicates. The shaded area represents ± 0.1 units around pH 8.0.

Supplementary Fig. 5

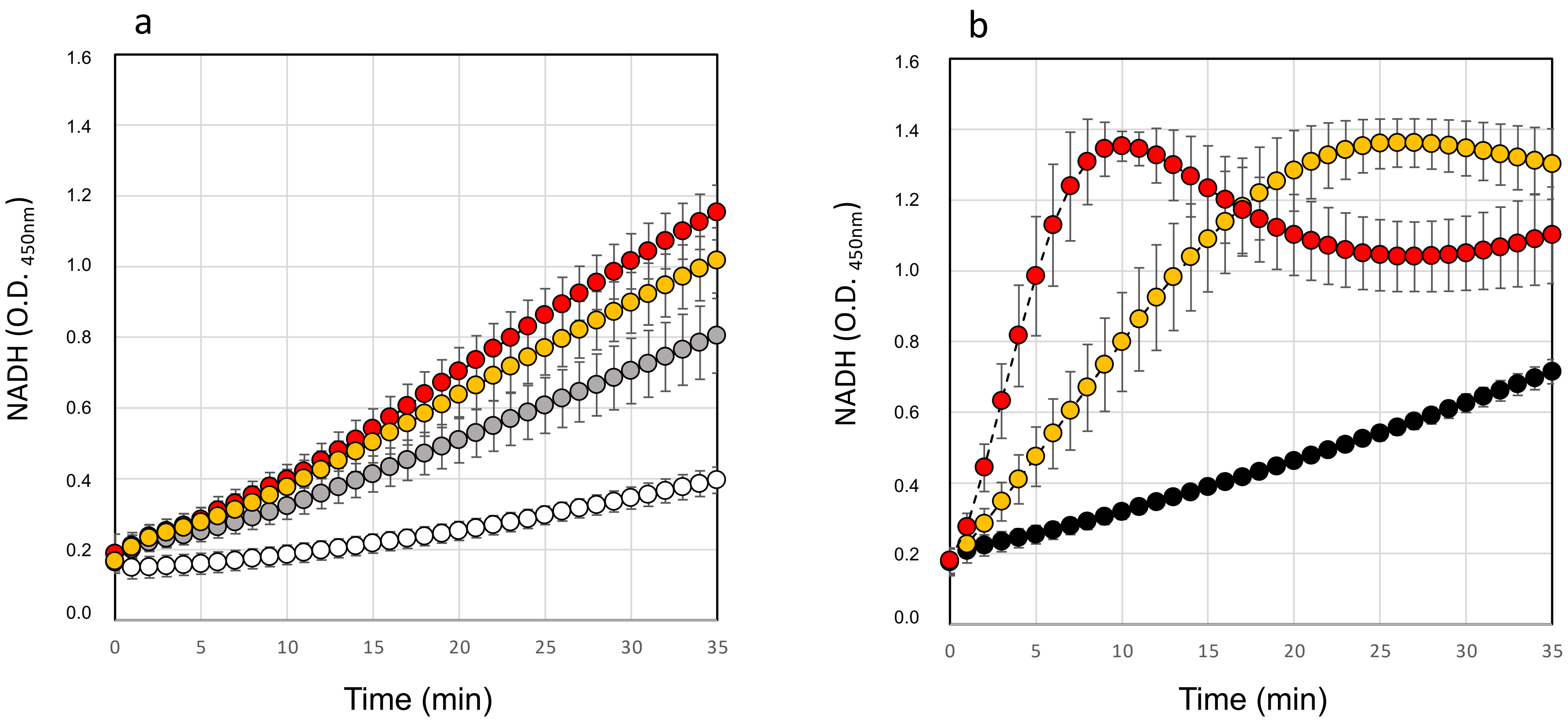

Phosphofructokinase (PFK) activity reported as the kinetic of NADH development according to PFK Activity Colorimetric Assay Kit (Sigma-Aldrich, Milan, Italy) using **a)** total cell extracts of EdCs of *S. thermophilus* and **b)** purified PFK of *Bacillus* sp. provided as positive control in PFK Activity Colorimetric Assay Kit (black circles). PFK activity was measured without (grey circles) or with the supplementation of 0.5 mM NH_3 (yellow circles) or 0.5 mM NH_4Cl (red circles). Blank sample (open circles) was prepared using total cell extracts of *S. thermophilus* EdCs plus all the reagents requested by the PFK Activity Colorimetric Assay Kit, except for PFK substrate. Data are reported as the average of five replicates. Error bars represent the standard deviation.
